# The gut-kidney axis in urolithiasis: roles of gut microbiota, metabolites, and therapeutic implications

**DOI:** 10.3389/fmicb.2025.1655808

**Published:** 2025-10-10

**Authors:** Dong Li, Zehong Li, Wei Liu

**Affiliations:** Department of Urology Surgery, The Third Affiliated Hospital of Gansu University of Chinese Medicine, Baiyin, China

**Keywords:** gut-kidney axis, urolithiasis, gut microbiota, oxalate metabolism, short-chain fatty acids, probiotics, prebiotics, fecal microbiota transplantation

## Abstract

Urolithiasis affects 2–20% of the global population and recurs frequently. Emerging evidence positions the gut–kidney axis as a central driver of stone formation. This review synthesizes epidemiological data, comparative metagenomic analyzes, and mechanistic studies to demonstrate that stone formers exhibit reduced *α*-diversity, depletion of oxalate-degrading taxa (e.g., *Oxalobacter*, *Lactobacillus*, *Bifidobacterium*), and enrichment of pro-inflammatory genera (*Escherichia*, *Bacteroides*). Microbial metabolites—oxalate, short-chain fatty acids, p-cresol, and secondary bile acids—modulate intestinal oxalate transport, systemic inflammation, and renal crystal nucleation. Therapeutic modulation via targeted probiotics, prebiotics, engineered Lactobacillus, or fecal microbiota transplantation restores oxalate homeostasis and attenuates nephrolithiasis in rodent models; however, human efficacy remains preliminary. Large-scale multi-omics cohorts and randomized controlled intervention trials are imperative to translate gut-centric strategies into precision urology.

## 1 Introduction

Urolithiasis is a widespread disease, affecting 2–20% of the global population (Kachkoul et al., 2023). Its prevalence is on the rise, closely following socioeconomic development (Kachkoul et al., 2023). Geographical and racial variations are notable; for instance, in Sub-Saharan Africa, the pooled prevalence among hospital—visiting patients was 9.4% (95% CI = 4.9–14%) (Kassaw et al., 2024). In Asia, the prevalence ranges from 1 to 19.1% across different regions (Liu et al., 2018). Calcium—type stones, especially calcium oxalate, are the most common, accounting for over 80% of cases (Kachkoul et al., 2023). The global burden of urolithiasis, as measured by incidence and disability-adjusted life—years, has shown a decrease in high Socio-Demographic Index countries but an increase in low and low-middle Socio-Demographic Index countries from 1990 to 2019 (Li et al., 2023). Calcium-type stones, particularly calcium oxalate, account for over 80% of cases, underscoring their predominant role in the disease landscape (Nazarian et al., 2025). The recurrence rate of urolithiasis is also a critical concern, with studies indicating that approximately 50% of patients experience at least one recurrence within five to ten years. This recurring nature not only exacerbates the physical suffering of patients but also imposes a substantial economic burden on healthcare systems, necessitating frequent medical interventions, hospitalizations, and long-term monitoring (Tamborino et al., 2024; Karagöz et al., 2022).

The etiology of urolithiasis is multifactorial, with traditional risk factors playing a pivotal role. Dietary habits are among the most well-established contributors (Siener et al., 2023). A diet rich in oxalate, sodium, and animal protein, coupled with inadequate fluid intake, significantly elevates the risk of stone formation. For instance, excessive consumption of oxalate-containing foods such as spinach, nuts, and rhubarb can increase urinary oxalate levels, promoting the precipitation of calcium oxalate crystals (Yang et al., 2024). Similarly, a high-sodium diet can enhance urinary calcium excretion, fostering stone development (Odermatt, 2011). Metabolic abnormalities, including hypercalciuria, hyperoxaluria, and hypocitraturia, are also well-documented risk factors. These imbalances disrupt the delicate equilibrium of urinary components, favoring crystal nucleation and growth (Owino et al., 2023). Genetic predisposition further complicates the risk profile, with monogenic diseases accounting for a higher proportion of stone formers in children and adolescents (Monico and Milliner, 2011). Additionally, obesity, diabetes, and metabolic syndrome have been increasingly linked to urolithiasis. Obesity, for example, may alter urinary acidification and increase urinary calcium excretion, thereby promoting stone formation (Trinchieri et al., 2017). However, despite our understanding of these traditional risk factors, their individual contributions and complex interactions in the pathogenesis of urolithiasis remain areas of active investigation and are not yet fully elucidated.

Recent research has spotlighted the gut-kidney axis as a novel and promising avenue for understanding and managing urolithiasis (Gao et al., 2022). This axis encapsulates the intricate two-way communication between the gut microbiota and the kidneys (Rui-Zhi et al., 2020). In the context of urolithiasis, the gut microbiota emerges as a key player that can influence stone formation through multiple pathways. First and foremost, gut microbes are intricately involved in oxalate metabolism. Certain bacteria, such as *Oxalobacter formigenes*, possess the unique ability to degrade oxalate in the gut (Stanford et al., 2020). When the population of these beneficial bacteria dwindles, the absorption of dietary oxalate may increase, subsequently raising urinary oxalate levels and the risk of calcium oxalate stone formation (Stanford et al., 2020). Recent reviews and conference data (2024–2025) consolidate the view that food-grade *Lactobacillus* and *Bifidobacterium* isolates behave as “generalist oxalotrophs” which constitutively express oxalyl-CoA decarboxylase (oxc) and formyl-CoA transferase (frc), enzymes that cleave dietary oxalate into CO₂ and formate within the intestinal lumen. Al-Kabe and Niamah (2024) surveyed global starter-culture research and noted that over 70% of tested *Lactobacillus* spp. and all *B. longum* subsp. infantis strains reduced soluble oxalate by >50% in fermented soymilk within 24 h, an activity that correlated with transcriptional up-regulation of oxc and concomitant lowering of luminal oxalate absorption in a Caco-2 transport model. Expanding these findings, the same group screened 42 Iraqi dairy-derived lactobacilli and reported in 2025 that *L. plantarum* IQ-6 and *L. paracasei* IQ-15 combined (10⁹ CFU/day) decreased urinary oxalate excretion by 32% in a rat hyperoxaluric model, while maintaining high gastrointestinal survival (pH 3, 0.3% bile) and full antibiotic susceptibility profiles compatible with probiotic criteria. Collectively, these works corroborate earlier metagenomic observations that enrichment of *Lactobacillus/Bifidobacterium* communities is inversely associated with 24 h urinary oxalate in human stone formers and provide mechanistic proof that live biotherapeutics targeting oxalate breakdown remain a feasible preventive strategy against calcium oxalate nephrolithiasis (Al-Kabe and Niamah, 2025). Beyond direct oxalate metabolism, the gut microbiota can indirectly impact urolithiasis via metabolic byproducts. For example, the production of short-chain fatty acids (SCFAs) through the fermentation of dietary fibers can influence systemic inflammation and immune responses. These effects, in turn, may create a conducive environment for stone formation within the urinary tract (Hong et al., 2023). Furthermore, the gut microbiota plays a crucial role in immune modulation. Dysbiosis, or imbalance, of the gut microbiota can lead to an aberrant immune response. This may trigger chronic low-grade inflammation in the urinary tract, promoting the aggregation of crystal-forming substances and the development of stones (Levy et al., 2017). Despite these emerging insights, our understanding of the gut-kidney axis in urolithiasis remains incomplete, offering a fertile ground for further exploration.

The gut-kidney axis represents a complex interplay between the gut microbiota and the kidneys. In urolithiasis, this axis has gained significant attention. Gut microbiota can influence urolithiasis through various mechanisms, including direct oxalate metabolism, indirect metabolic pathways, and immune modulation (Tsuji et al., 2024). Dysbiosis of the gut microbiota may lead to an increase in uremic toxins, which can affect renal function and contribute to stone formation. This review aims to synthesize the latest evidence on the gut microbiota’s roles in urolithiasis, exploring both direct and indirect mechanisms and evaluating their therapeutic potential. By bridging the gap between experimental findings and clinical practice, we aspire to facilitate the development of innovative approaches that can ultimately improve patient outcomes and reduce the burden of urolithiasis on healthcare systems globally.

## 2 Gut microbiota and urolithiasis

The complex relationship between the gut microbiota and urolithiasis has been gradually unveiled in recent research. The gut microbiota, a vital community of microorganisms in the gastrointestinal tract, is now recognized as a significant factor influencing stone formation (Yuan et al., 2023). Understanding this relationship is key to exploring novel therapeutic approaches for urolithiasis.

### 2.1 Composition and function of gut microbiota

The gut microbiota is a complex ecological community comprised of trillions of microorganisms, including bacteria, fungi, viruses, and other microbes, with bacteria being the most predominant and extensively studied component (Vemuri et al., 2020). These microorganisms primarily reside in the large intestine and are distributed across different phyla, with the most common ones being *Firmicutes*, *Bacteroidetes*, *Actinobacteria*, and *Proteobacteria*. The gut microbiota composition varies among individuals due to factors such as genetics, diet, age, lifestyle, and antibiotic use, and it can also change over time within the same individual (Vemuri et al., 2020).

The gut microbiota plays a crucial role in maintaining host health through various functions. In terms of nutrition metabolism, it helps in the breakdown of complex carbohydrates, proteins, and lipids that are otherwise indigestible by human enzymes (Valdes et al., 2018). For instance, gut bacteria ferment dietary fiber into SCFAs like acetate, propionate, and butyrate, which serve as energy sources for colonocytes and other cells in the body. These SCFAs also have anti-inflammatory properties and contribute to the maintenance of gut barrier function (Ramos Meyers et al., 2022). Additionally, the gut microbiota is involved in the synthesis of certain vitamins, such as vitamin K and some B vitamins, which are essential for various physiological processes (Ellis et al., 2021).

The gut microbiota plays a vital role in the development and regulation of the host immune system. It interacts with immune cells in the gut-associated lymphoid tissue, helping to shape the maturation of immune cells and modulating immune responses (Yoo et al., 2020). A healthy gut microbiota promotes the production of antibodies and cytokines, which are crucial for defending against pathogens and maintaining immune homeostasis (Gomaa, 2020). Furthermore, the gut microbiota contributes to the maintenance of gut barrier function by stimulating the production of mucus and tight junction proteins, thereby preventing the translocation of harmful substances and pathogens into the systemic circulation (Di Vincenzo et al., 2024).

### 2.2 Differences in gut microbiota between stone formers and healthy individuals

Numerous studies have compared the gut microbiota composition of individuals with urolithiasis and healthy controls, revealing significant differences (Ticinesi et al., 2018; Zampini et al., 2019; Lee et al., 2025). Overall, stone formers tend to exhibit reduced gut microbiota diversity compared to healthy individuals. A lower diversity of gut microbiota is often associated with dysbiosis, a state of microbial imbalance that can disrupt the normal functioning of the gut ecosystem and contribute to various diseases (Al et al., 2023).

**Figure 1:**
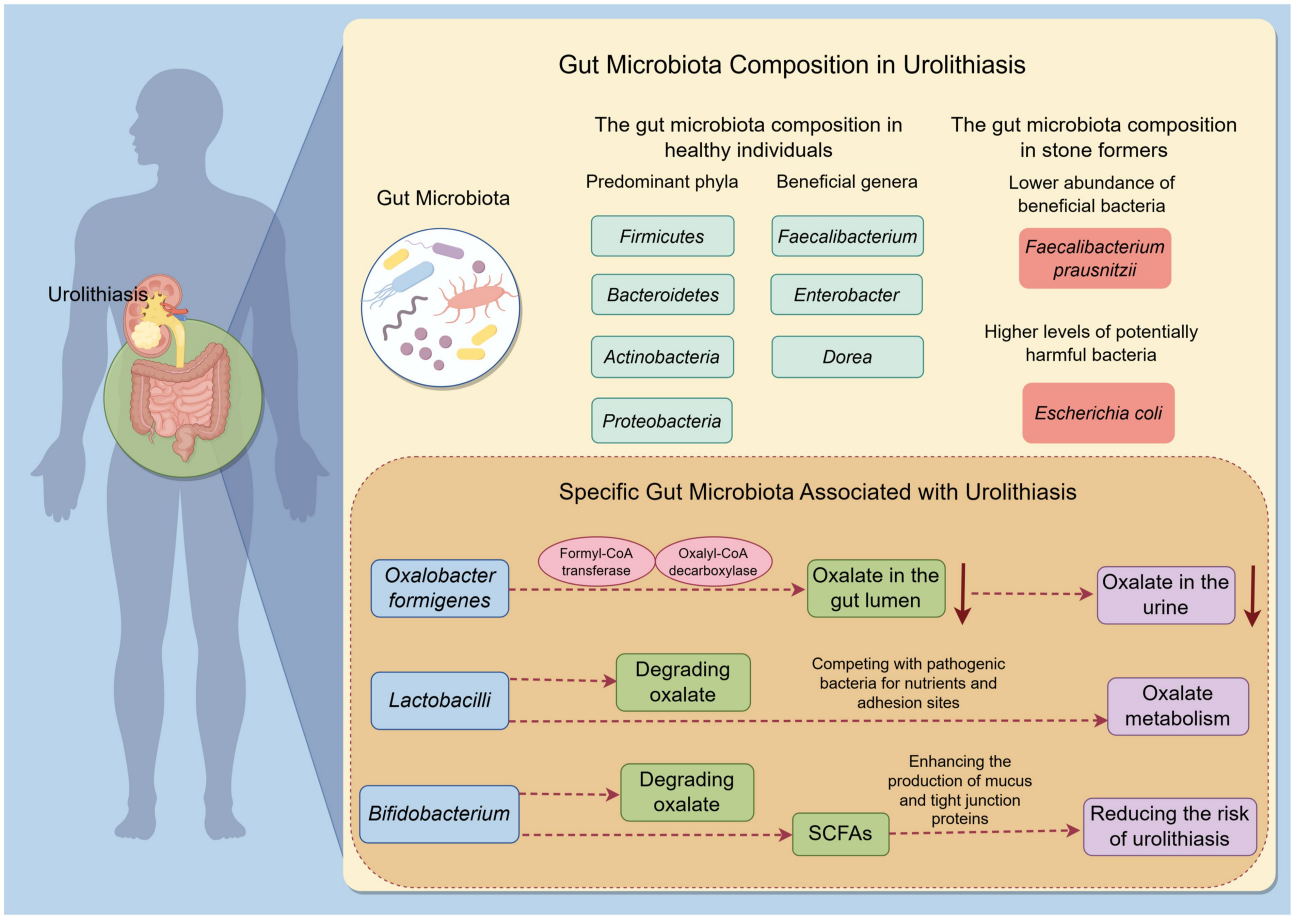
Gut microbiota composition and functional differences in urolithiasis by Figdraw. This figure illustrates the differences in gut microbiota composition and function between stone formers and healthy individuals. It highlights the reduced diversity and specific alterations in beneficial and potentially harmful bacteria in stone formers. The figure also emphasizes the functional differences related to oxalate metabolism.

At the phylum level, some studies have reported alterations in the relative abundances of *Firmicutes* and *Bacteroidetes* in stone formers (Liu et al., 2023). For example, a meta-analysis by Yuan et al. found that patients with urolithiasis had a higher abundance of *Bacteroides* and *Escherichia*, while *Prevotella* was more abundant in healthy controls. These shifts in phylum-level composition may reflect broader changes in the gut microbial community structure that are linked to stone formation (Yuan et al., 2023). At the genus level, differences are also evident. Stone formers often show a reduced abundance of certain beneficial bacteria, such as *Faecalibacterium*, *Enterobacter*, and *Dorea. Faecalibacterium prausnitzii*, a well-known anti-inflammatory bacterium that produces butyrate, is typically depleted in stone formers. In contrast, potentially pathogenic or pro-inflammatory bacteria, such as *Escherichia coli* and other *Enterobacteriaceae* members, may be enriched in the gut microbiota of stone formers (Stern et al., 2016) (Figure 1).

Moreover, specific functional alterations related to oxalate metabolism have been observed in the gut microbiota of stone formers. Metagenomics studies have revealed that stone formers have a lower representation of genes associated with oxalate degradation compared to healthy individuals (Al et al., 2023). This reduction in oxalate-metabolizing capacity may lead to increased oxalate absorption in the gut and subsequently higher urinary oxalate excretion, which is a known risk factor for calcium oxalate stone formation.

### 2.3 Specific gut microbiota associated with urolithiasis

*Oxalobacter formigenes*, a gram-negative, anaerobic bacterium, has a unique capability to degrade oxalate. It utilizes oxalate as its sole carbon and energy source through the action of two key enzymes: formyl-CoA transferase and oxalyl-CoA decarboxylase. By degrading oxalate in the gut lumen, this bacterium reduces the amount of oxalate available for absorption into the bloodstream and subsequent excretion in urine (Miller and Dearing, 2013) (Figure 1). A meta-analysis found that the presence of *Oxalobacter formigenes* was associated with a lower risk of calcium oxalate stone formation (Kachroo et al., 2021). However, its role in stone formation remains somewhat controversial. Some studies have shown that not all individuals colonized with *Oxalobacter formigenes* are protected from stone formation, suggesting that other factors and microbes also play important roles in this process (Miller and Dearing, 2013).

*Lactobacilli* are a genus of gram-positive, facultative anaerobic bacteria commonly found in the human gut. Some species of *Lactobacilli* have been shown to possess oxalate-degrading activity (Turroni et al., 2007). For example, *Lactobacillus acidophilus* and *Lacticaseibacillus casei* have demonstrated the ability to degrade oxalate *in vitro*. *Lactobacilli* may also influence oxalate metabolism indirectly by competing with pathogenic bacteria for nutrients and adhesion sites in the gut, thereby maintaining a healthy gut microbiota balance (Di Cerbo et al., 2016). Additionally, *Lactobacilli* are known for their immunomodulatory effects. They can enhance the production of anti-inflammatory cytokines and strengthen the gut barrier function. By reducing inflammation and oxidative stress in the gut and kidneys, Lactobacilli may contribute to a lower risk of stone formation (Di Cerbo et al., 2016) (Figure 1).

*Bifidobacterium* is another genus of beneficial gut bacteria that have been implicated in oxalate metabolism. Certain Bifidobacterium species, such as *Bifidobacterium bifidum* and *Bifidobacterium longum*, have shown oxalate-degrading capability. *Bifidobacterium* also plays a significant role in maintaining gut barrier function. They produce SCFAs that nourish colonocytes and enhance the production of mucus and tight junction proteins (O'Callaghan and van Sinderen, 2016). Furthermore, *Bifidobacterium* has immunomodulatory properties, helping to regulate immune responses and reduce inflammation in the gut. A healthy population of *Bifidobacterium* in the gut may thus help prevent the translocation of harmful substances and pathogens into the systemic circulation, which could have indirect beneficial effects on kidney health and reduce the risk of urolithiasis (Gavzy et al., 2023) (Figure 1).

## 3 Metabolites in the gut-kidney axis and urolithiasis

The metabolites produced by the gut microbiota serve as crucial mediators in the gut-kidney axis and play a central role in urolithiasis pathogenesis. These metabolites, such as oxalate and SCFAs, directly impact renal crystal formation and inflammation (Mehta et al., 2016). Investigating their mechanisms of action and therapeutic potential offers promising avenues for managing urolithiasis (Table 1).

**Table 1:** Gut microbiota-derived metabolites in urolithiasis: mechanisms and therapeutic implications.

Metabolite class	Key microbes	Mechanism	Ref.
Oxalate	Functional microbial communities	More effective induction of oxalate metabolism than functional microbial species	Miller et al. (2017)
Oxalate	Multi-species bacterial network	Inhibit urinary stone disease and ensure healthy oxalate homeostasis	Miller et al. (2019)
Oxalate	Gut microbiota	Affect the formation of calcium oxalate renal calculi caused by high daily tea consumption	Chen et al. (2021)
SCFAs	Gut microbiota	Regulate oxalate metabolism and affect renal calcium oxalate stones disease	Liu et al. (2020)
Oxalate	Gut microbiota	Hyperoxaluria causes gut microbiota dysbiosis and enriches oxalate—metabolizing bacterial species	Agudelo et al. (2024)
Gut microbiota	FMT	Alter urine chemistry and reduce stone-forming risk factors	Hunthai et al. (2024)
SCFAs	Probiotic Lactiplantibacillus plantarum N−1	Prevent ethylene glycol—induced kidney stones by regulating gut microbiota and enhancing intestinal barrier function	Wei et al. (2021)
SCFAs	Short-Chain Fatty Acids	Prevent glyoxylate—induced calcium oxalate stones via a GPR43—dependent immunomodulatory mechanism	Jin et al. (2021)
SCFAs	Short-Chain Fatty Acids	Reduce renal calcium oxalate stones by regulating intestinal oxalate transporter SLC26A6	Liu et al. (2021)
Amines and phenols	p-cresol	Activate the NLRP3 inflammasome, leading to renal inflammation and fibrosis	Suttapitugsakul et al. (2025)
Bile acids	Gut microbiota	Influence calcium and oxalate metabolism by binding to and inhibiting intestinal oxalate—binding proteins	Zhou et al. (2023)

SCFAs, short-chain fatty acids; FMT, fecal microbiota transplantation; NLRP3, NOD-like receptor family pyrin domain containing 3.

### 3.1 Oxalate metabolism

Oxalate is a key component in the formation of calcium oxalate stones. The gut plays a crucial role in oxalate metabolism. Dietary oxalate is absorbed in the intestine, and the gut microbiota can degrade oxalate, thereby reducing its absorption and systemic exposure. The gut microbiota’s role in oxalate metabolism extends beyond *Oxalobacter formigenes* (Falony, 2018). Miller et al. found that functional microbial communities are more effective than individual functional microbial species in inducing oxalate metabolism *in vivo*, suggesting that a diverse gut microbiota may have a more significant impact on oxalate homeostasis (Miller et al., 2017). Additionally, Miller et al. showed that a multi-species bacterial network can inhibit urinary stone disease and maintain healthy oxalate homeostasis (Miller et al., 2019). Chen et al. also demonstrated that gut microbiota can affect the formation of calcium oxalate renal calculi caused by high daily tea consumption (Chen et al., 2021).

In patients with urolithiasis, gut microbiota dysbiosis may lead to reduced oxalate-degrading capacity, resulting in increased oxalate absorption and elevated urinary oxalate levels, which in turn increase the risk of calcium oxalate stone formation. Liu et al. explored the relationship between gut microbiota and short-chain fatty acids in renal calcium oxalate stone disease and found that gut microbiota dysbiosis is associated with altered oxalate metabolism (Liu et al., 2020). Agudelo et al. discovered that hyperoxaluria can cause gut microbiota dysbiosis and selectively enrich oxalate—metabolizing bacterial species in recurrent kidney stone formers (Agudelo et al., 2024).

Probiotics or prebiotics may modulate the gut microbiota to enhance oxalate degradation. A randomized, controlled trial demonstrated that lactic acid bacteria could reduce idiopathic hyperoxaluria (Liu et al., 2020). Fecal microbiota transplantation (FMT) has also shown potential in modifying urine chemistry risk factors for urinary stone disease. Hunthai et al. (2024) found that FMT could alter urine chemistry and reduce stone-forming risk factors. However, further research is needed to determine the optimal probiotics, prebiotics, and FMT protocols for preventing and treating urolithiasis.

### 3.2 Short-chain fatty acids

SCFAs, such as acetate, propionate, and butyrate, are produced by the gut microbiota through the fermentation of dietary fiber. These SCFAs have various physiological functions, including regulating gut barrier function, immune responses, and energy metabolism (Mukhopadhya and Louis, 2025). Multiple studies have shown that SCFAs can enhance gut barrier function by promoting the synthesis of mucin and tight junction proteins, thereby preventing the translocation of bacterial products and toxins into the bloodstream (Xiao et al., 2024; Liu et al., 2021). This is significant for urolithiasis, as gut barrier dysfunction may allow bacterial products and toxins to enter the systemic circulation, potentially inducing systemic inflammation and oxidative stress, which contribute to kidney stone formation. Wei et al. found that probiotic Lactiplantibacillus plantarum N-1 can prevent ethylene glycol—induced kidney stones by regulating gut microbiota and enhancing intestinal barrier function (Wei et al., 2021). Other research has also demonstrated the role of SCFAs in maintaining gut integrity and preventing inflammatory conditions that may contribute to stone formation (Tian et al., 2022).

SCFAs play a role in immune regulation by modulating the differentiation and function of immune cells, such as T regulatory cells and macrophages. They can reduce inflammation in the gut and systemically. Inflammation is a key factor in kidney stone formation, as it can promote crystal adhesion and aggregation. Jin et al. showed that short-chain fatty acids can prevent glyoxylate—induced calcium oxalate stones via a GPR43—dependent immunomodulatory mechanism (Jin et al., 2021). Butyrate, as an anti-inflammatory substance, may indirectly affect kidney stone formation by influencing gut inflammation. SCFAs can also regulate the expression of intestinal oxalate transporters, such as SLC26A6, thereby affecting oxalate absorption and excretion. Liu et al. found that short-chain fatty acids reduce renal calcium oxalate stones by regulating the expression of intestinal oxalate transporter SLC26A6 (Liu et al., 2021).

However, the specific mechanisms by which SCFAs regulate gut barrier function, immune responses, and energy metabolism in the context of urolithiasis require further investigation. Additionally, factors such as the types and doses of SCFAs, as well as individual differences in gut microbiota, may influence their effects on urolithiasis.

### 3.3 Other metabolites

Besides oxalate and SCFAs, the gut microbiota produces various other metabolites, such as amines, phenols, and bile acids, which may also impact kidney function and urolithiasis (Mukhopadhya and Louis, 2025). Amines and phenols, such as p-cresol and indoxyl sulfate, are derived from the gut microbiota’s metabolism of proteins and amino acids. These metabolites can be absorbed into the bloodstream and excreted in urine (McFarlane et al., 2022). At high concentrations, they may induce oxidative stress and inflammation in the kidneys, promoting kidney stone formation. Suttapitugsakul et al. found that p-cresol can activate the NOD-like receptor family pyrin domain containing 3 (NLRP3) inflammasome, leading to renal inflammation and fibrosis, which may increase the risk of kidney stones (Suttapitugsakul et al., 2025). Other study has also highlighted the role of these metabolites in inducing oxidative stress and inflammation in renal tubular epithelial cells, contributing to kidney damage and stone formation (Tian et al., 2025).

Bile acids, synthesized by the liver and further metabolized by gut microbiota, play a role in lipid digestion and absorption. They can also act as signaling molecules to regulate glucose and lipid metabolism. Recent a study suggest that bile acids may influence calcium and oxalate metabolism. For example, secondary bile acids can bind to and inhibit the activity of intestinal oxalate—binding proteins, reducing oxalate absorption (Zhou et al., 2023). However, the specific mechanisms by which bile acids affect urolithiasis remain unclear and warrant further exploration. Research in this area is ongoing to better understand the complex interactions between gut microbiota—derived metabolites and their effects on kidney stone formation.

In conclusion, gut microbiota metabolites play a crucial role in the gut-kidney axis and urolithiasis. Oxalate metabolism, SCFAs, and other metabolites collectively influence kidney stone formation through multiple mechanisms. However, current research on the role of gut microbiota metabolites in urolithiasis is still limited, with many mechanisms remaining to be elucidated. Future studies should focus on employing multi-omics technologies to comprehensively analyze the types and functions of gut microbiota metabolites in urolithiasis, revealing their metabolic networks and regulatory mechanisms. Conducting clinical trials to evaluate the efficacy and safety of probiotics, prebiotics, FMT, and other interventions in modulating gut microbiota metabolites for urolithiasis prevention and treatment is also essential. Exploring the potential of gut microbiota metabolites as biomarkers for urolithiasis diagnosis and prognosis, as well as investigating the interactions between gut microbiota metabolites and host genetics, diet, and lifestyle factors to develop personalized prevention and treatment strategies for urolithiasis, are promising research directions. By strengthening research in these areas, we aim to deepen our understanding of the role of gut microbiota metabolites in urolithiasis and promote the translation of research findings into clinical applications, ultimately improving the quality of life for patients with urolithiasis.

## 4 Mechanisms linking gut microbiota, metabolites, and urolithiasis

The gut microbiota and its metabolites interact through various mechanisms to influence urolithiasis. These include direct effects on oxalate metabolism and indirect effects via inflammation and immunity (Kim et al., 2022). Additionally, the gut microbiota’s impact on gut barrier function plays a significant role in stone formation. Understanding these intricate mechanisms is essential for developing targeted therapeutic strategies.

### 4.1 Direct effects

**Figure 2:**
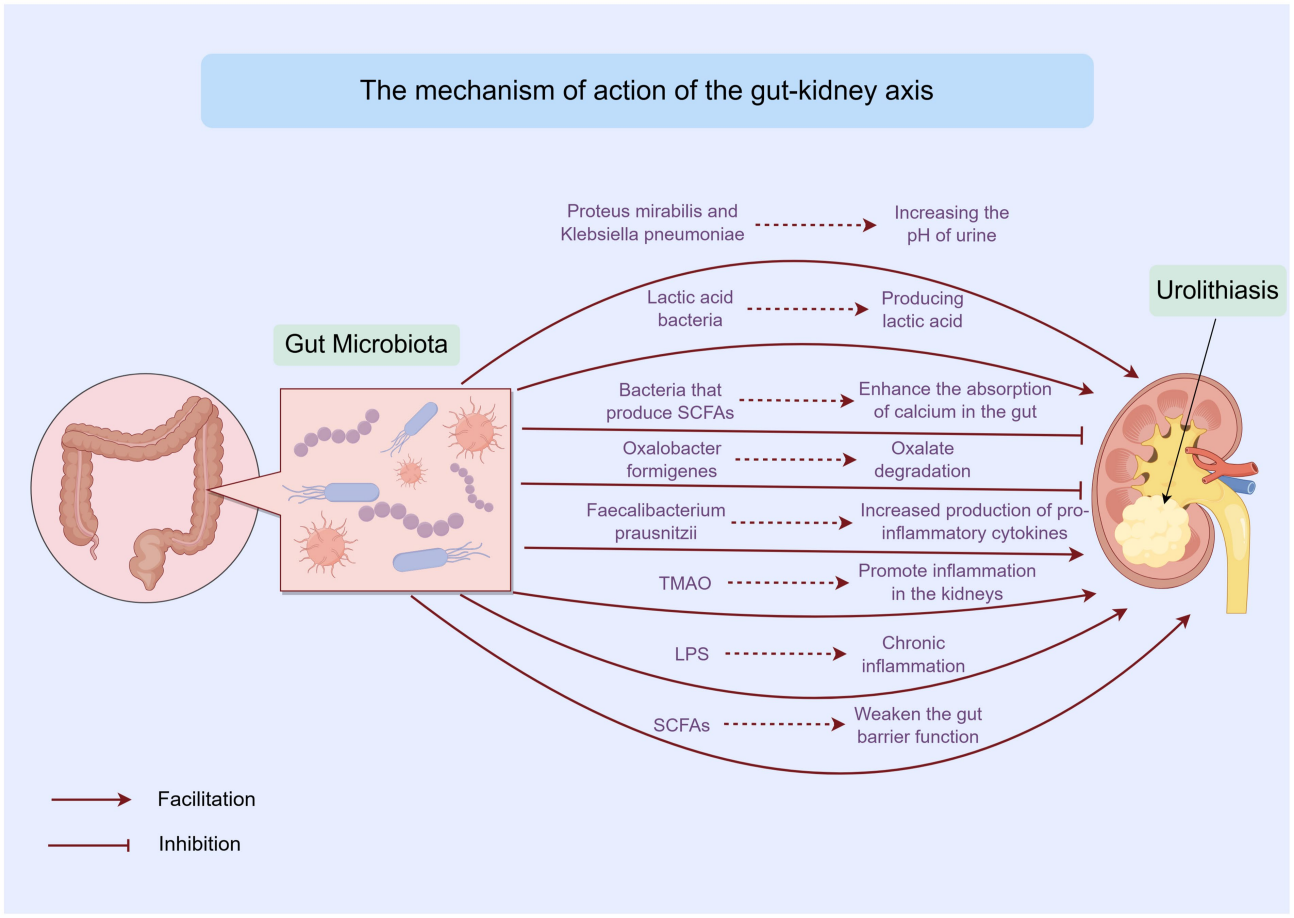
Mechanisms linking gut microbiota, metabolites, and urolithiasis by Figdraw. This figure outlines the direct and indirect mechanisms through which gut microbiota and its metabolites influence urolithiasis. It includes the impact of oxalate metabolism, SCFAs, and other metabolites on kidney stone formation.

The gut microbiota can directly influence urolithiasis through various mechanisms. Certain bacteria possess the ability to degrade oxalate, a key component in calcium oxalate stones, which account for approximately 75% of kidney stones (Hunthai et al., 2024). *Oxalobacter formigenes*, a gram-negative anaerobic bacterium, specializes in oxalate degradation using formyl-CoA transferase and oxalyl-CoA decarboxylase. Its presence in the gut reduces the amount of oxalate available for absorption and subsequent urinary excretion (Chmiel et al., 2022). However, trials involving oxalate-degrading probiotics, such as those containing *Oxalobacter formigenes*, have not produced definitive findings. One major factor contributing to this limitation is the high sensitivity of *Oxalobacter formigenes* to antibiotics, which are commonly used in clinical settings (Lange et al., 2012) (Figure 2). Besides, some studies have shown that not all individuals colonized with *Oxalobacter formigenes* are protected from stone formation, suggesting that other factors and microbes also play important roles in this process.

In addition to oxalate-degrading bacteria, some gut bacteria can affect the physical and chemical properties of urine, thereby influencing stone formation and growth. For instance, certain bacteria can alter the pH of urine. Urease-producing bacteria, such as *Proteus mirabilis* and *Klebsiella pneumoniae*, can hydrolyze urea into ammonia, increasing the pH of urine. This makes the urine more alkaline, which may promote the formation of struvite stones (Jung et al., 2023). Conversely, certain lactic acid bacteria are capable of producing lactic acid, which reduces the pH of the intestinal environment and may potentially influence the absorption and excretion of urinary stone components (Wan et al., 2024).

Moreover, gut bacteria can also influence the concentration of substances in urine. Some bacteria can affect the absorption of minerals and nutrients in the gut, thereby altering the levels of these substances in the blood and urine. For example, bacteria that produce SCFAs can enhance the absorption of calcium in the gut. This may reduce the amount of calcium available to combine with oxalate in the urine, thus lowering the risk of calcium oxalate stone formation (Ticinesi et al., 2018).

### 4.2 Indirect effects via inflammation and immunity

Growing evidence suggests that the gut microbiota can modulate systemic inflammation and immune responses, which in turn affect kidney health and stone formation. The gut microbiota plays a vital role in maintaining the balance of the immune system. When the gut microbiota is dysbiotic, it can lead to an imbalance in the production of pro-inflammatory and anti-inflammatory cytokines (Wastyk et al., 2021; De Luca et al., 2025) (Figure 2). For example, a decrease in beneficial bacteria such as *Faecalibacterium prausnitzii*, which has anti-inflammatory properties, can result in increased production of pro-inflammatory cytokines like interleukin-6 (IL-6) and tumor necrosis factor-alpha (TNF-*α*). These cytokines can enter the bloodstream and reach the kidneys, promoting inflammation in the renal tissues and creating a favorable environment for stone formation (Yuan et al., 2023).

The production of certain metabolites by the gut microbiota can also influence inflammation and immune responses. For instance, trimethylamine N-oxide (TMAO), a metabolite derived from dietary choline and L-carnitine, has been associated with an increased risk of cardiovascular disease and chronic kidney disease. Elevated levels of TMAO may promote inflammation in the kidneys and affect the metabolism of urinary stone components, thereby contributing to stone formation (Capolongo et al., 2023). Additionally, lipopolysaccharides (LPS), which are components of the outer membrane of gram-negative bacteria, can trigger strong immune responses when they enter the bloodstream. Dysbiosis of the gut microbiota can increase the permeability of the gut barrier, allowing more LPS to translocate into the systemic circulation. This can lead to chronic inflammation, which may exacerbate kidney damage and increase the risk of urolithiasis (Wei et al., 2021) (Figure 2).

The gut microbiota can also influence the function of immune cells. Regulatory T cells (Tregs), which play a crucial role in maintaining immune tolerance and suppressing excessive immune responses, can be modulated by certain gut bacteria (Bhutta et al., 2024). A decrease in the abundance of Tregs due to gut microbiota dysbiosis may result in unchecked immune responses in the kidneys, leading to tissue damage and the promotion of stone formation. Furthermore, the gut microbiota can affect the maturation and function of dendritic cells, which are important antigen-presenting cells. Dysregulated dendritic cell activity can lead to abnormal activation of T cells and B cells, contributing to autoimmune and inflammatory responses in the kidneys and other organs (Wilson et al., 2022).

Recent evidence demonstrates that *Bifidobacterium longum* subsp. infantis Iraq-Basrah 3-derived exopolysaccharides (EPS) significantly attenuate LPS-induced IL-6 and TNF-*α* secretion in THP-1 macrophages, highlighting the immunomodulatory potential of specific commensal strains (Hussein et al., 2025). This finding is particularly relevant to urolithiasis, as elevated IL-6 and TNF-α promote renal tubular epithelial–mesenchymal transition and osteogenic differentiation, thereby facilitating Randall plaque formation and calcium oxalate nucleation (Suttapitugsakul et al., 2025). Furthermore, the EPS from *B. longum* subsp. infantis enhances regulatory T-cell (Treg) expansion while suppressing Th17 responses via TLR2/MyD88-dependent signaling, a pathway that has been independently shown to reduce renal calcium oxalate crystal deposition in murine models (Jin et al., 2021). Consequently, depletion of Bifidobacterium spp. in stone formers—consistently reported in 16S rRNA meta-analyze—may remove a critical brake on IL-17–driven neutrophil recruitment and subsequent reactive oxygen species (ROS)-mediated crystal adhesion to renal epithelia (Yuan et al., 2023). Collectively, these data position commensal-derived EPS as candidate microbe-based therapeutics capable of restoring the Treg/Th17 balance and mitigating inflammation-facilitated stone formation.

### 4.3 Interaction with the gut barrier function

The gut barrier function is essential for maintaining the integrity of the gut and preventing the translocation of harmful substances and pathogens into the systemic circulation. The gut microbiota plays a crucial role in maintaining the gut barrier function (Barbara et al., 2021). Beneficial bacteria such as *Lactobacilli* and *Bifidobacterium* can enhance the production of mucus and tight junction proteins, thereby strengthening the gut barrier. They also compete with pathogenic bacteria for adhesion sites and nutrients, preventing the overgrowth of harmful microbes that can disrupt the gut barrier (Segui-Perez et al., 2025) (Figure 2).

When the gut microbiota is dysbiotic, the gut barrier function can become compromised. This can lead to increased gut permeability, allowing bacteria, bacterial fragments, and metabolites to enter the bloodstream (Dmytriv et al., 2024). These substances can trigger systemic inflammation and immune responses, which may have detrimental effects on kidney health. For example, the translocation of bacterial endotoxins such as LPS can activate immune cells in the kidneys, leading to the production of inflammatory mediators and oxidative stress (Sun et al., 2022; Zhong et al., 2020). This can result in kidney damage and promote the formation of stones. Furthermore, the increased absorption of oxalate due to impaired gut barrier function can elevate urinary oxalate levels, increasing the risk of calcium oxalate stone formation.

In addition to the direct effects of gut microbiota on the gut barrier, certain metabolites produced by the gut microbiota can also influence gut barrier function. SCFAs, such as butyrate, propionate, and acetate, are important metabolites that help maintain the integrity of the gut barrier. Butyrate, in particular, serves as the primary energy source for colonocytes and promotes the production of mucus and tight junction proteins (Liang et al., 2022; Gasaly et al., 2021). A decrease in the production of SCFAs due to gut microbiota dysbiosis can weaken the gut barrier function, making the gut more susceptible to inflammation and the translocation of harmful substances (Yang et al., 2020; Pohl et al., 2022). This can ultimately affect kidney health and contribute to the development of urolithiasis (Figure 2).

Overall, the gut microbiota and its metabolites are intricately linked to urolithiasis through a variety of direct and indirect mechanisms. Understanding these complex interactions can provide valuable insights into the pathogenesis of urolithiasis and pave the way for the development of novel therapeutic strategies targeting the gut microbiota and its metabolites. However, further research is needed to fully elucidate these mechanisms and to overcome the limitations of current studies, such as small sample sizes and confounding factors, in order to establish more effective and targeted interventions for the prevention and treatment of urolithiasis.

## 5 Therapeutic implications of gut microbiota modulation

Modulating the gut microbiota presents a novel therapeutic approach for urolithiasis prevention and treatment. Probiotics, prebiotics, FMT, and dietary interventions have shown potential in altering the gut microbiota composition and function (Jung et al., 2023; Zheng and Cao, 2024) (Table 2). Exploring their efficacy and safety in clinical settings is crucial for translating these findings into effective clinical applications.

**Table 2:** Gut microbiota modulation for urolithiasis therapy.

Study type	Subjects	Intervention	Mechanism	Key findings	Ref.
Analytical	Commercial probiotics	Checked active *Oxalobacter formigenes*	Balanced gut microbiota, enhanced oxalate degradation	Active *Oxalobacter formigenes* found in some probiotics	Ellis et al. (2015)
Experimental	Hyperoxaluric rats	Engineered *Lactobacillus plantarum* with oxalate decarboxylase	Genetically modified probiotics for oxalate degradation	Lowered gut oxalate, cut kidney stone risk	Paul et al. (2018)
Clinical (pilot)	Patients post-endourological surgery	Potassium citrate, magnesium and probiotics supplement	Stimulated beneficial bacteria growth	Reduced crystalluria	Vittori et al. (2022)
Basic	Dysbiotic gut microbiota animal model	Lactiplantibacillus plantarum	Modulated gut arginine metabolism	Less renal Calcium oxalate stones via metabolic changes	Liu et al. (2021)
Analytical	Hyperoxaluric rats	Tested Lactobacillus paragasseri and Lacticaseibacillus paracasei	Specific probiotics broke down gut oxalate	Reduced renal oxalate, lowered stone risk	Mehra et al. (2022)
Metabolomic	Oxalate—degrading *Lactobacillus acidophilus* and *Lactobacillus gasseri*	Metabolomic profiling	Explored metabolic features of probiotics	Identified unique metabolic traits for stone prevention	Chamberlain et al. (2019)
Basic	Rat kidney stone model	FMT	Transplanted healthy gut microbiota	Altered gut microbiota composition, cut stone-forming factors	Hunthai et al. (2024)
Analytical	Gut microbiota of oxalate stone formers	Studied functional eubacteria species	Explored roles of specific microbes	Linked certain bacteria to stone formation	Suryavanshi et al. (2018)
Experimental	Rat model	Combined oxalate—degrading bacteria and herbal extract	Combined microbial and plant—based approach	Reduced urinary oxalate, new treatment strategy	Afkari et al. (2019)
System modeling	Gut microbiota in oxalate homeostasis	Complex system modeling	Showed diverse bacteria drove oxalate homeostasis	Stressed gut microbiota diversity for stone prevention	Mukherjee et al. (2025)
Basic	Calcium oxalate crystal—induced kidney injury model	Rutin intervention	Anti-oxidative stress and gut flora modulation	Rutin reduced kidney injury and stone risk	Zhang et al. (2025)
Basic	EG-induced Calcium oxalate nephrolithiasis rat model	*Quercus dentata* Thunb. leaves extract	Inhibited Calcium oxalate crystallization, regulated OPN/CD44 and NLRP3 pathways	Reduced Calcium oxalate stones via crystallization inhibition and pathway modulation	Zhang et al. (2025)
Mechanistic	Glyoxylate—induced Calcium oxalate stone model	SCFAs	GPR43—dependent immunomodulation	SCFAs prevented stone formation via immune regulation	Jin et al. (2021)

FMT, fecal microbiota transplantation; RCTs, randomized controlled trials; SCFAs, short-chain fatty acids; Slc26a6, solute carrier family 26 member 6; Nlrp3, NOD-like receptor family pyrin domain containing 3.

### 5.1 Probiotics and prebiotics

Probiotics and prebiotics have emerged as promising therapeutic options for urolithiasis by modulating the gut microbiota. Probiotics can restore the balance of the gut microbiota and enhance the degradation of oxalate (Taheri et al., 2024; Wigner et al., 2022). For instance, Ellis et al. (2015) found viable *Oxalobacter formigenes* in some commercial kidney stone probiotic supplements. Paul et al. (2018) developed a designer probiotic *Lactobacillus plantarum* expressing oxalate decarboxylase. Prebiotics can selectively stimulate the growth of beneficial bacteria. Vittori et al. (2022) reported that food supplementation based on potassium citrate, magnesium, and probiotics reduced crystalluria in patients undergoing endourological surgery for stone disease. However, more research is needed to determine the optimal strains, dosages, and treatment durations for urolithiasis.

Liu et al. (2021) demonstrated that Lactiplantibacillus plantarum could reduce renal calcium oxalate stones by regulating arginine metabolism in the gut microbiota. Wei et al. (2021) showed that probiotic *Lactiplantibacillus* plantarum N-1 could prevent ethylene glycol-induced kidney stones by regulating gut microbiota and enhancing intestinal barrier function. Mehra et al. (2022) analyzed and characterized *Lactobacillus paragasseri* and *Lacticaseibacillus paracasei.* Chamberlain et al. (2019) conducted metabolomic profiling of oxalate-degrading probiotic *Lactobacillus acidophilus* and *Lactobacillus gasseri*.

### 5.2 FMT

FMT involves transferring the gut microbiota from a healthy donor to a patient and could potentially re-establish a healthy gut microbiota composition in urolithiasis patients. Hunthai et al. (2024) explored the role of FMT in a rat model of kidney stone disease and found that it altered the gut microbiota composition, suggesting a potential therapeutic effect. By introducing a diverse and healthy gut microbiota, FMT may help reduce the production of uremic toxins and restore normal metabolic processes related to stone formation. However, challenges such as donor selection, standardization of the procedure, and potential risks of infection need to be addressed before its widespread use in urolithiasis treatment. Suryavanshi et al. (2018) studied functional eubacteria species along with trans-domain gut inhabitants and their role in oxalate stone disease. Afkari et al. (2019) used a combination of oxalate-degrading bacteria and herbal extract to reduce urinary oxalate in a rat model.

### 5.3 Dietary interventions

Dietary interventions play a significant role in modulating the gut microbiota and preventing urolithiasis. A diet rich in fiber can promote the growth of beneficial gut bacteria and increase the production of SCFAs. For example, increasing the intake of fruits, vegetables, and whole grains can provide prebiotic substrates for the gut microbiota. Mukherjee et al. (2025) revealed that oxalate homeostasis is driven by diverse oxalate-degrading bacteria, highlighting the importance of dietary choices in managing gut microbiota for stone prevention. Additionally, reducing the intake of high-oxalate foods, such as spinach, rhubarb, and nuts, can lower the risk of hyperoxaluria. In patients with autosomal dominant polycystic kidney disease, dietary regimens like caloric restriction, intermittent fasting, and ketogenic diets are being investigated for their potential to slow disease progression, which may also be relevant to urolithiasis.

Zhang et al. (2025) showed that rutin ameliorated calcium oxalate crystal-induced kidney injury through anti-oxidative stress and modulation of intestinal flora. Zhang et al. (2025) highlighted the potential of *Quercus dentata* Thunb. leaves extract in inhibiting Calcium oxalate crystallization and ameliorating ethylene glycol-induced Calcium oxalate kidney stones via the OPN/CD44 and NLRP3 pathways. Chen et al. (2021) suggested that gut microbiota affect the formation of calcium oxalate renal calculi caused by high daily tea consumption. Jin et al. (2021) found that SCFAs could prevent glyoxylate-induced calcium oxalate stones by GPR43-dependent immunomodulatory mechanisms.

In conclusion, modulation of the gut microbiota through probiotics, prebiotics, FMT, and dietary interventions offers novel therapeutic approaches for urolithiasis. However, further research is needed to optimize these strategies and overcome existing limitations.

## 6 Research limitations and future directions

Despite the valuable insights gained from current research on the gut-kidney axis in urolithiasis, several limitations exist. These include small sample sizes, heterogeneous study populations, and methodological inconsistencies. Addressing these limitations through large-scale cohort studies, multi-omics integration, and well-designed intervention trials will enhance our understanding and pave the way for more effective therapeutic strategies.

### 6.1 Limitations in study designs and methodologies

The research on the gut-kidney axis in urolithiasis faces several limitations that may impact the reliability and generalizability of the findings. One major issue is the small sample size in many studies, which can compromise statistical power and make it harder to detect significant differences between groups (Yuan et al., 2023). This also limits the ability to perform subgroup analyzes, which could provide insights into the role of factors such as age, sex, and genetic background.

The heterogeneity of study populations is another challenge. Urolithiasis patients are diverse in terms of stone composition, metabolic abnormalities, and geographical and racial backgrounds (Reed and Hsi, 2024). However, many studies do not adequately account for this diversity, raising questions about the broad applicability of the findings. This heterogeneity can also introduce confounding variables that may obscure the true relationship between gut microbiota and urolithiasis. The majority of studies are observational, which makes it difficult to establish causality. Observational studies can identify associations between gut microbiota and urolithiasis, but they cannot determine whether changes in gut microbiota directly influence stone formation or if these changes are merely secondary effects of the disease. The reliance on self-reported dietary and lifestyle data in these studies also increases the risk of recall bias and inaccuracies.

The dynamic nature of the gut microbiota poses additional methodological challenges. The composition of gut microbiota can vary significantly over time due to factors such as diet, stress, and antibiotic use (Zhang et al., 2022). However, many studies only provide a single snapshot of the gut microbiota, failing to capture its temporal variability. This can lead to an incomplete understanding of how changes in gut microbiota over time contribute to the development and progression of urolithiasis. Moreover, the lack of standardized methodologies for assessing gut microbiota can affect the comparability and replicability of results. Differences in sample collection, processing, and analysis techniques can lead to inconsistencies in findings (Ducarmon et al., 2020). For example, variations in DNA extraction methods, sequencing platforms, and bioinformatics pipelines can significantly impact the identification and quantification of microbial species (Lim et al., 2018).

### 6.2 Future research directions

To deepen our understanding of the gut-kidney axis in urolithiasis and develop effective therapeutic strategies, several future research directions should be explored. Large-scale cohort studies are necessary to address the limitations of small sample sizes and heterogeneity (Yuan et al., 2023; Kachroo et al., 2021). These studies should include diverse urolithiasis patient populations, covering different stone compositions, metabolic profiles, and demographic backgrounds (Reed and Hsi, 2024; Liu et al., 2023). Longitudinal follow-up of these cohorts can enhance our understanding of the temporal relationships between gut microbiota changes and stone formation. Establishing standardized protocols for data collection and analysis will ensure consistency across studies.

The integration of multi-omics technologies offers a powerful approach to comprehensively analyze the gut microbiota and its metabolites in urolithiasis. Metagenomics can reveal the genetic potential of microbial communities, while metatranscriptomics and metaproteomics can shed light on their functional activity (Liu et al., 2023; Zhang et al., 2023). Metabolomics can identify and quantify the small molecules produced by gut microbiota, which may serve as intermediates in the gut-kidney axis. This integrated analysis can help identify key microbial functions and metabolic pathways involved in stone formation and may uncover new therapeutic targets (Gao et al., 2022).

Well-designed intervention studies are crucial to establish causality and evaluate the efficacy of gut microbiota modulation in preventing and treating urolithiasis. Randomized controlled trials (RCTs) of probiotics, prebiotics, and FMT should be conducted with clearly defined endpoints, such as changes in urinary stone risk factors, stone recurrence rates, and patient-reported outcomes. Mechanistic studies are also needed to clarify how gut microbiota and its metabolites influence urolithiasis. Animal models can be useful for studying these mechanisms in a controlled setting, but it is important to ensure that findings are translatable to humans.

Personalized treatment approaches based on individual gut microbiota profiles represent a promising avenue for future research. By understanding how variations in gut microbiota composition and function influence an individual’s risk of urolithiasis and response to treatment, clinicians can tailor interventions to meet each patient’s specific needs. This may involve the use of precision medicine strategies, such as microbiota-based biomarkers for disease stratification and treatment selection. Furthermore, further exploration of the interactions between gut microbiota, host genetics, diet, and lifestyle factors are essential to fully understand the complex etiology of urolithiasis. These interactions likely play a significant role in modulating the gut-kidney axis and may provide opportunities for developing more effective prevention and treatment strategies that consider the whole-person context.

In summary, while existing research on the gut-kidney axis in urolithiasis has provided valuable insights, there are limitations that need to be addressed. By conducting large-scale cohort studies, utilizing multi-omics technologies, performing well-designed intervention trials, pursuing personalized treatment approaches, and investigating the interplay of multiple factors, we can enhance our understanding of this complex relationship and ultimately improve the clinical management of urolithiasis.

## 7 Conclusion

The gut-kidney axis represents a pivotal mechanism in the pathogenesis of urolithiasis, with gut microbiota and their metabolites playing central roles in modulating stone formation. Dysbiosis contributes to elevated urinary oxalate, systemic inflammation, and impaired gut barrier function, thereby promoting calcium oxalate crystallization. Microbiota-derived metabolites, including short-chain fatty acids and oxalate-degrading agents, are key mediators in these processes. Emerging therapeutic strategies, such as probiotics, prebiotics, and fecal microbiota transplantation, offer promising avenues for prevention and treatment. However, further large-scale clinical trials and mechanistic studies are essential to validate efficacy and optimize protocols. Integration of multi-omics approaches and personalized interventions based on individual microbiota profiles may enhance therapeutic outcomes. Continued research is warranted to translate these findings into clinical practice and reduce the global burden of urolithiasis.
